# Radiative cooling induced coherent maser emission in relativistic plasmas

**DOI:** 10.1126/sciadv.adt8912

**Published:** 2025-04-11

**Authors:** J. Bilbao, Thales Silva, Luís O. Silva

**Affiliations:** GoLP/Instituto de Plasmas e Fusão Nuclear, Instituto Superior Técnico, Universidade de Lisboa, 1049-001 Lisboa, Portugal.

## Abstract

Relativistic plasmas in strong electromagnetic fields exhibit distinct properties compared to classical plasmas. In astrophysical environments, such as neutron stars, white dwarfs, active galactic nuclei, and shocks, relativistic plasmas are pervasive and are expected to play a crucial role in the dynamics of these systems. Despite their significance, experimental and theoretical studies of these plasmas have been limited. Here, we present the first ab initio high-resolution kinetic simulations of relativistic plasmas undergoing synchrotron cooling in a highly magnetized medium. Our results demonstrate that these plasmas spontaneously generate coherent linearly polarized radiation in a wide range of parameters via the electron cyclotron maser instability, with radiative losses altering the saturation mechanism. Thus, the plasma continuously amplify coherent radiation for substantially longer durations of time. These findings highlight fundamental differences in the behavior of relativistic plasmas in strongly magnetized environments and align with astronomical phenomena, such as pulsar emission and fast radio bursts.

## Teaser

Strongly magnetized relativistic plasmas spontaneously amplify coherent radiation, offering insights into astronomical signatures.

## INTRODUCTION

Relativistic plasmas are expected to arise around neutron stars, black holes, and other compact objects through various mechanisms, e.g., pair cascades or the Schwinger mechanism (*1*–*5*), and in laboratory experiments, e.g., with intense lasers or relativistic particle beams (*6*–*12*). These highly energetic plasmas, with electron energies comparable or higher than the rest mass of the particle, form in environments with intense electromagnetic fields, which can sometimes approach the electric and magnetic Schwinger limit ($E_{\text{Sc}}≃1.3\times 10^{18}\;V/m$ and $B_{\text{Sc}}≃4.4\times 10^{9}\;T$, respectively) (*13*–*17*). Under these conditions, quantum electrodynamical (QED) processes, such as nonlinear Breit-Wheeler, Compton scattering, and radiation reaction, are dominant or comparable to classical plasma processes. Therefore, phenomena such as turbulence (*18*–*20*), shock formation (*21*, *22*), laser-plasma interactions (*11*, *13*, *14*, *23*), beam-plasma interactions (*24*), and, as shown here, kinetic instabilities will exhibit substantial quantitative differences from their classical plasma counterparts and also manifest qualitatively distinct behaviors and features (*25*).

The complex nature of plasmas in extreme electromagnetic environments has driven substantial interest in investigating their kinetic properties. Even simplified electromagnetic field configurations show rich phenomena and can yield unexpected results. For instance, plasmas undergoing strong synchrotron cooling have been shown to develop an anisotropic ring–shaped momentum distribution, characterized by a population inversion over the Landau levels, $\partial f/\partial p_{⊥}>0$, where *f* represents the plasma momentum distribution and $p_{⊥}$ is the momentum perpendicular to the magnetic field **B** (*26*–*29*). However, despite these advances, the collective properties of these strongly magnetized plasmas remain underexplored, particularly regarding the self-consistent electrodynamical effects. Theoretical and experimental investigations are needed to fully understand plasmas in this extreme regime.

One major challenge is that first-principles simulations are constrained by the vast separation of relevant spatial and temporal scales, which differ by several orders of magnitude. This requires using high resolutions over large simulation domains to capture all relevant physics, both spatially and temporally. The computational resources necessary to perform these numerical simulations, including all the relevant physics, have only recently become available. Replicating these extreme conditions in laboratory experiments is also highly challenging, and only recent advancements in experimental techniques have made it possible to generate plasmas under these extreme environments (*9*, *10*, *12*).

In this study, we address this gap by conducting the first and largest-scale, first-principles numerical simulations that demonstrate how relativistic plasmas embedded in strong magnetic fields can spontaneously produce linearly polarized coherent radiation via the electron cyclotron maser instability (ECMI) (*30*–*33*). We demonstrate how the instability is qualitatively modified by the inclusion of synchrotron losses. This phenomenon occurs in collisionless plasmas with relativistic temperatures, where radiation reaction plays a crucial role. Our simulations reveal that synchrotron cooling first establishes the Landau population inversion, in the shape of a ring momentum distribution, and then maintains it for longer timescales than previously thought possible, leading to continuous coherent amplification of radiation and a modified saturation state of the maser instability.

## RESULTS

**Fig. 1.**
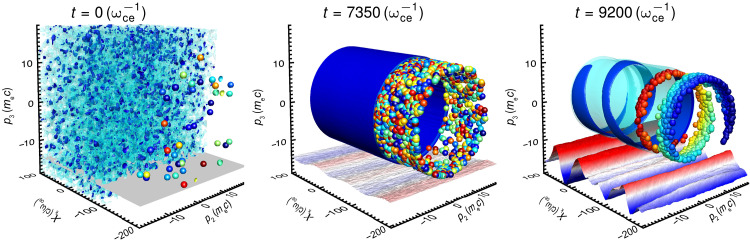
Self-consistent PIC simulations demonstrate the onset of ECMI and coherent amplification of radiation. The temporal evolution (from left to right) of the plasma distribution function f(r,p,t) shown as a three-dimensional (3D) projection and $p_{2}-p_{3}-x_{k}$ of the 6D phase space, where $p_{2}$ and $p_{3}$ are the momentum directions perpendicular to the *B* field, with $B/B_{\text{Sc}}=0.002$, and $x_{k}$ is the spatial direction along the propagation of the X-mode (also perpendicular to *B*). Two isosurfaces of the distribution function are represented: The light-blue and blue surfaces are at 0.5 and 0.8 of the peak value of the distribution function, respectively. The projection in the bottom plane (red-blue colors) represents the normalized amplitude of electric field associated with the amplified electromagnetic wave (X-mode). A sample of the plasma particles is also shown, with the color representing their azimuthal phase (between 0 and 2π, from red to blue) with respect to the amplified electromagnetic wave. Left: Initially, the plasma consists of a Maxwellian thermal plasma with initial momentum distribution $f_{0}\propto e^{-{|p|}^{2}/\left(2p_{\text{th}}^{2}\right)}$, where $p_{\text{th}}=1000\;m_{e}c$, no X-mode is observed, and the particles have random phases. Middle: A ring momentum distribution function has developed because of synchrotron cooling and amplification of the X-mode begins, but particles are still arranged in random phases. Right: The amplified X-mode is evident, and the ring is azimuthally bunched along the direction of propagation of the X-mode, as seen by the spiral structure in phase space, and the clear phase alignment, as seen by the color of the particles and matching of the wave phase with the phase-space structure, following the corresponding color scale.

We investigated, through large-scale, high-resolution particle-in-cell (PIC) simulations (see Materials and Methods), tenuous pair plasmas embedded in strong magnetic fields, where the cyclotron frequency $\omega_{\text{ce}}=\mathrm{eB}/m_{e}$ is much larger than the plasma frequency $\omega_{\text{pe}}=\sqrt{n_{e}e^{2}/\left(\varepsilon_{0}m_{e}\right)}$, where e and $m_{e}$ are the electron charge and electron mass, respectively; $n_{e}$ is the pair plasma density; and $\varepsilon_{0}$ is the permittivity of free space. To the best of our knowledge, these simulations represent the most extensive study in terms of both spatial and temporal scales for this system to date (see Materials and Methods). PIC simulation results, shown in Fig. 1, illustrate that an initial thermal plasma in its proper rest frame evolves into a ring momentum distribution characterized by steep gradients in the perpendicular momentum component $p_{⊥}$ with respect to the *B* field and a narrow energy spread $\Delta p_{⊥}$. This evolution triggers the efficient onset of the ECMI, which coherently amplifies electromagnetic thermal fluctuations in the magnetized plasma, generating X-mode electromagnetic waves. This process leads to phase trapping by the amplified wave, a characteristic signature of the instability, as confirmed by the phase-space projection after the onset of the instability, shown in Fig. 1.

The X-mode ECMI arises from a plasma characterized by a population inversion, i.e., $\partial f/\partial p_{⊥}>0$, and a narrow spread in $p_{∥}$. Electrons gyrate in the plane perpendicular to the magnetic field and interact with X-modes, which are linearly polarized electromagnetic waves whose wave vector and electric field are perpendicular to the guiding magnetic field, i.e., k⊥B, E⊥B, and k⊥E. Depending on whether the electrons rotate slightly faster or slower than the wave phase, they are accelerated or decelerated; consequently, their gyrofrequency ($\omega_{\text{ce}}/\gamma$, where γ is the Lorentz factor of the particle) decreases or increases, respectively. For the wave to gain energy from the particles, more electrons must have gyrofrequencies (or its harmonics) slightly below the wave frequency ($\omega ≳n\omega_{\text{ce}}/\gamma$). This requires $\partial f/\partial p_{⊥}>0$, ensuring that more particles transfer energy to the wave than they extract from it. The energy transfer amplifies the interacting X-mode waves at the fundamental and harmonics of the electron gyration frequency within the population inversion through phase trapping. As the wave is amplified, more electrons are phase trapped, generating an unstable feedback (*30*, *34*). The ring-shaped momentum distribution is an ideal candidate to drive the X-mode ECMI.

The evolution of the synchrotron-cooled plasma, the subsequent growth rate of the X-mode, and the dynamical timescales of the instability, as seen in the simulations, can be directly computed from kinetic theory with the inclusion of radiative losses (*26*–*28*, *35*, *36*). For a tenuous pair plasma in a strong magnetic field, the momentum distribution of a plasma evolves as $f\left(p_{⊥},p_{∥},\tau \right)=f\left(\frac{p_{⊥}}{1-\tau p_{⊥}},\frac{p_{∥}}{1-\tau p_{⊥}}\right)/{\left(1-\tau p_{⊥}\right)}^{4}$, where $p_{⊥}$ is normalized to $m_{e}c$, τ is a normalized time such that $\tau =\frac{2\alpha }{3}B_{0}\omega_{\text{ce}}t$, where $B_{0}=B/B_{\text{Sc}}$ and α is the fine-structure constant (*26*). An important feature is that *f* is bounded between $0<p_{⊥}<1/\left(2\alpha B_{0}\omega_{\mathrm{ce}}t\right)$ and that the resulting ring radius asymptotically approaches the boundary at $p_{R}=3/\left(2\alpha B_{0}\omega_{\text{ce}}t\right)$ (*28*). Thus, a relativistic plasma, independently of the initial shape of *f*, will develop into an anisotropic ring momentum distribution (*26*–*29*). This results from the nonlinear nature of synchrotron radiation, which bunches the momentum distribution in the radial momentum direction $p_{⊥}$. The radiation reaction force violates the conservation of phase-space volume, in contrast with the classical collisionless plasma dynamics mediated by the Lorentz force (*37*, *38*). Therefore, synchrotron radiation drives the plasma away from kinetic equilibrium and eventually fulfils the conditions for efficient maser emission.

The growth rate of X-modes, Γ, as a function of *t* and the wave angular frequency ω can be approximated using the Wentzel–Kramers–Brillouin (WKB) approximation, which, for $\omega_{\text{ce}}\gg \omega_{\text{pe}}$, simplifies to $d\Gamma /\mathrm{dt}\ll \omega^{2}$ (*39*) and yields

$$\Gamma \left(\omega ,t\right)=2\pi^{2}\frac{\omega_{\text{pe}}^{2}}{\omega }\sum_{n=1}^{\infty }\left\{p_{⊥}^{\prime 2}{\frac{\partial f_{⊥}\left(t\right)}{\partial p_{⊥}}|}_{p_{⊥}=p_{⊥}^{\prime }}{\left[J_{n}^{\prime }\left(\frac{\omega p_{⊥}^{\prime }}{n\omega_{\text{ce}}}\right)\right]}^{2}\right\}$$ (1)

where $J_{n}^{\prime }\left(b\right)$ is the first derivative of the *n*th-order Bessel function evaluated at *b*, $p_{⊥}^{\prime }=\sqrt{n^{2}\omega_{\text{ce}}^{2}/\omega^{2}-1}$ is the resonant momentum with the given frequency ω, $f_{⊥}\left(p_{⊥},t\right)$ is the perpendicular momentum distribution, i.e., integrated along the magnetic field direction $f_{⊥}\left(p_{⊥},t\right)=\int_{-\infty }^{\infty }f\left(p_{⊥},p_{∥},t\right)\partial p_{∥}$. Initially, Γ changes rapidly, as the cooling process is fast because of the initial thermal spread. As the plasma cools, *f* changes slower, and $d\Gamma /\mathrm{dt}\propto d\left(\partial f/\partial p_{⊥}\right)/\mathrm{dt}$ decreases accordingly. This gradual slowing of the cooling process at lower values of $p_{⊥}$ ultimately ensures the validity of the WKB approximation. Equation 1 shows some of the key universal emission features characteristic of the synchrotron-induced electron cyclotron maser: (i) The amplification rate is proportional to the plasma density $\omega_{\text{pe}}^{2}\propto n_{e}$, (ii) occurs near the different cyclotron harmonic resonances $\omega_{\text{ce}}/\gamma$, and (iii) is proportional to $\partial f_{⊥}/\partial p_{⊥}$.

As the distribution function cools down, it develops a small region between the ring radius and the edge of momentum space, of width $\Delta p≃3\sqrt{3}/{\left(2\alpha B_{0}p_{\text{th}}\omega_{\text{ce}}t\right)}^{2}$, where $p_{\text{th}}$ is the initial thermal spread. The width of the ring allows to estimate the maximum growth rate at the fundamental frequency as $\Gamma_{\text{max}}≃18\pi \frac{\omega_{\text{pe}}^{2}}{\omega_{\text{ce}}}\frac{p_{R}\gamma_{R}}{\Delta p^{2}}\left[J_{1}^{\prime }\left(1\right)\right]2$, using the relativistic approximation ($\gamma_{R}∼p_{R}$), the growth rate estimate simplifies to $\Gamma_{\text{max}}≃2{\pi \alpha }^{2}B_{0}^{2}p_{\text{th}}^{2}\omega_{\text{pe}}^{2}\omega_{\text{ce}}t^{2}$, and $\Gamma_{\text{max}}$ shows how an initial plasma with a higher thermal spread $p_{\text{th}}$ leads to a higher growth rate and how the growth rate increases with time due to the distribution *f* developing a steeper gradient, as the plasma cools.

**Fig. 2.**
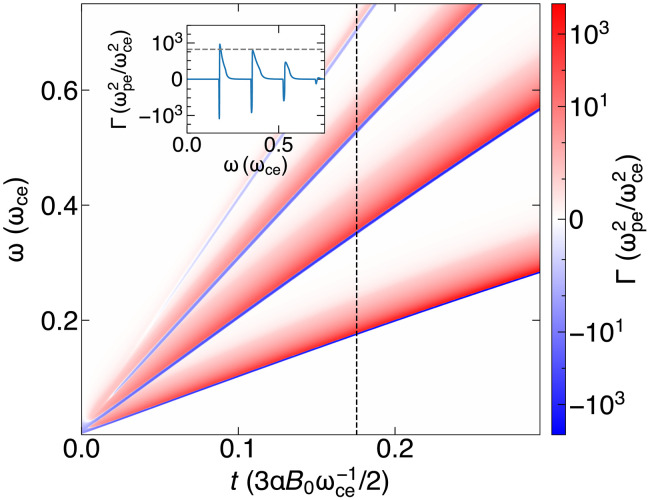
The key emission features of the cyclotron maser instability are demonstrated by temporal evolution of the X-mode growth rate, Γ(ω,t), as described by Eq. 1, for a typical distribution function, $f_{⊥}\left(p_{⊥},t=0\right)=e^{-p_{⊥}^{2}/\left(2p_{\text{th}}^{2}\right)}/p_{\text{th}}^{3}{\left(2\pi \right)}^{1/2}$, where $p_{\text{th}}=100\;m_{e}c$. Since the ring distribution is a general characteristic of hot plasmas (i.e., those with $p_{\text{th}}\gg m_{e}c$) undergoing synchrotron cooling, this initial distribution function effectively represents the qualitative behavior of maser emission under various initial conditions (*26*, *28*). The figure demonstrates that the emission is evenly spaced in ω space, as evident from the line out of Γ(ω,t=0.165τ) shown in the inset, which highlights the emission and absorption regions, where Γ>0 and Γ<0, respectively. The emission predominantly occurs near the harmonics of the resonant frequency, which gradually converge toward the harmonics of $\omega_{\text{ce}}$, as the ring distribution cools down and asymptotically approaches $p_{⊥}=0$.

To illustrate the properties of the ECMI with radiative cooling, we determine Γ (shown in Fig. 2) for an initial Maxwellian distribution (this greatly simplifies analytical calculations). The growth rate and dynamics of the ECMI do not significantly vary with the initial distribution shape (e.g., Maxwell-Jüttner), as plasmas with large energy spreads will always converge to a ring distribution (*26*, *28*).

The synchrotron-cooled plasma produces a small emission region in ω above the frequency $n\omega_{\text{ce}}/\gamma_{r}$ and a small absorption region below that frequency, for each harmonic *n*, as observed in Fig. 2. The emission changes with time due to the shifting resonance condition as the ring cools down, and the estimate for $\Gamma_{\text{max}}$ is confirmed (compare inset axis of Fig. 2).

For the efficient onset of the ECMI, two condtions must be met: (i) Γ>0 and (ii) the ring distribution provides several e-foldings to the interacting wave, before dephasing. Initially, the ring forms and develops $\partial f/\partial p_{⊥}>0$, within a small region of momentum space of width $\Delta p≃3\sqrt{3}/{\left(2\alpha B_{0}p_{\text{th}}^{2}\omega_{\text{ce}}t\right)}^{2}$ below the ring radius. At that point, waves with ω in resonance within that region of momentum space are amplified at a rate comparable to $\Gamma_{\text{max}}$. This amplification continues for a short-time interval $\Delta t_{d}=3\sqrt{3}/\left(2\alpha B_{0}p_{\text{th}}^{2}\omega_{\text{ce}}\right)$ until the ring cools down enough that it dephases and ω is no longer in resonance. Now, the ring is in resonance with a slightly higher ω, and the process repeats itself.

Initially, the cooling rate is too rapid to efficiently amplify any frequency before the ring dephases, i.e., $\Delta t_{d}<\Gamma_{\text{max}}^{-1}$. As $\Gamma_{\text{max}}$ increases over time, eventually, $\Gamma_{\text{max}}^{-1}$ becomes smaller than $\Delta t_{d}$, allowing the ring to remain in resonance for several e-folding times and provide efficient amplification, which occurs, in the proper frame of the plasma, at time

$$t_{o}=\sqrt{\frac{1}{\sqrt{3}\pi \alpha }}\frac{{\mathrm{eB}}_{\text{Sc}}}{m_{e}\omega_{\text{pe}}}\sqrt{\frac{B_{0}}{p_{\text{th}}}}\omega_{\text{ce}}^{-1}$$ (2)

or $t_{o}\;\left(12\;\mu s\right)≃{\left[B\;\left(\text{MG}\right)\;n_{e}\;\left(10^{6}\;{\text{cm}}^{-3}\right)\;p_{\text{th}}\;\left(100\;m_{e}c\right)\right]}^{-1/2}$; $t_{o}$ determines the time at which coherent emission begins from the beginning of the cooling process. The scaling of Eq. 2 has been confirmed with PIC simulations (see the Supplementary Materials).

**Fig. 3.**
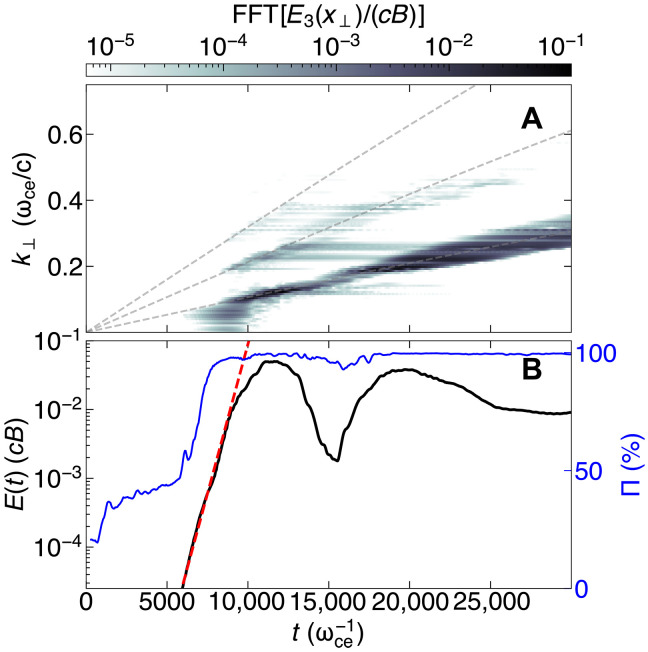
PIC simulations illustrate the temporal evolution of the X-mode electromagnetic spectrum and energy, which agree with the theoretical estimates, both qualitatively and quantitatively. (**A**) Fast Fourier transform (FFT) of the electric field component $E_{3}$, which is perpendicular to both the ambient magnetic field (of amplitude *B*) and the wave vector k. This spectrum shows the ongoing evolution of the electromagnetic fields during both the linear and nonlinear phases of the instability. Theoretical predictions for the maximum growth rates (i.e., the resonant frequencies) from Eq. 1 are overlaid as gray dashed lines. (**B**) Normalized time evolution of the electric field amplitude (black line) and the degree of polarization, derived from the Stokes parameters (blue line). The estimate for the linear growth rate Γ, obtained by numerically solving the full dispersion relation, is compared with the simulation results after the onset of the maser instability but still at early times ($t_{o}<t≲12,000\;\omega_{\text{ce}}^{-1}$), shown as a red dashed line, which follows the relation $E_{3}\propto e^{\Gamma t}$.

The electromagnetic spectrum of the amplified X-mode is shown in Fig. 3A and demonstrates that the X-mode spectrum peaks near the regions with the highest Γ, with the first three harmonics being amplified, whereas the fourth is much weaker, as analytically predicted (see Fig. 2). The degree of polarization, in Fig. 2B, demonstrates that the self-consistent radiation resulting from emission is highly linearly polarized. This is a result of the X-mode being the fastest-growing mode in highly magnetized plasmas (*40*).

The classical ECMI, a collisionless plasma process, reaches saturation due to the azimuthal phase trapping of the particles by the wave. As phase-space volume is conserved, when particles are azimuthally trapped, their momentum distribution must expand radially, broadening the ring distribution and reducing its gradient $\partial f/\partial p_{⊥}$. This eventually stabilizes the classical ECMI. Before saturation and for the electrons to provide energy to the wave, it is necessary that $\delta \omega =\omega -\omega_{\text{ce}}/\gamma ≳0$ (*30*). At saturation ($t∼1.2\times 10^{4}\;\omega_{\text{ce}}^{-1}$ in Fig. 3), particles overshoot the phase trapping condition and now δω≲0: Particles extract energy from the previously amplified wave. This is a well-known phenomenon (*30*), which explains the dip in electric field amplitude at $t∼1.5\times 10^{4}\;\omega_{\text{ce}}^{-1}$ in Fig. 3B and the saturated state, as shown in devices such as gyrotrons, free-electron-lasers, and ion-channel-lasers (*30*, *31*). PIC simulations capture the ECMI well beyond the linear regime and demonstrate that synchrotron-cooled plasmas evolve differently in the nonlinear stage of the instability, as seen in Fig. 3A: Unexpectedly, the spectrum continues to evolve with the X-mode spectrum shifting to higher frequencies and widening the spectrum. The emitted radiation is trapped in our simulation domain, mimicking an infinite plasma volume. For a finite volume plasma, the X-modes are expected to escape and convert to light waves.

The synchrotron-cooled ECMI introduces additional dynamics due to the effects of radiative losses, which do not conserve phase-space volume. As particles radiate synchrotron emission, they continue to bunch in the radial momentum direction. This creates a competition between azimuthal phase trapping, driven by the ECMI, which diffuses the gradient in $p_{⊥}$, and radiative losses, which continually attempt to bunch the distribution radially and sustain the gradient in $p_{⊥}$. The radiative losses are, thus, responsible for both establishing the initial ring distribution and maintaining it throughout the emission process.

**Fig. 4.**
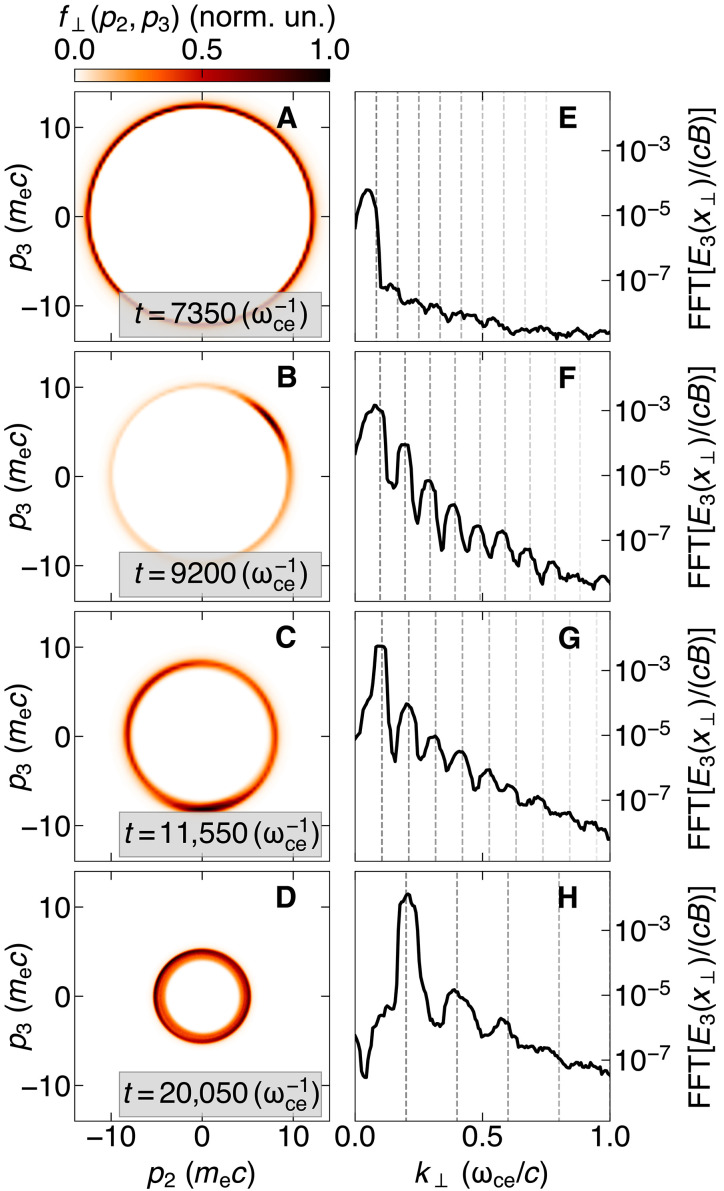
PIC simulations illustrate that ring distributions are sustained beyond the linear regime of the instability. This is seen in the evolution of a radiatively cooled electron-positron plasma undergoing the ECMI, starting from an initial Maxwellian distribution with $p_{\text{th}}=1000\;m_{e}c$. Column 1 (**A** to **D**) displays the electron momentum distribution integrated along the magnetic field direction $x_{1}$, $f_{⊥}\left(p_{2},p_{3}\right)$, while column 2 (**E** to **H**) shows the spectrum of the associated X-mode wave, with dashed lines indicating the expected harmonics, as predicted by our theoretical model. Each row corresponds to different times in the simulation: (A) and (E) shows the developed ring distribution from an initial Maxwellian plasma; (B) and (F) shows the onset of the ECMI, characterized by phase trapping (the positrons, not shown here, bunch up on the opposite phase of the ring), and narrow emission observed in the spectrum; (C) and (G) shows the widening of the ring distribution as the system reaches the standard ECMI saturation point; (D) and (H) demonstrates the further evolution of the distribution as the instability transitions to the nonlinear regime, where the ring widens and the emission is broader.

PIC simulations confirm this interplay, as demonstrated by the evolution of the perpendicular momentum distribution of the plasma, as it transitions from the linear to the nonlinear regime of the instability. After the establishment of the ring distribution with a narrow momentum spread (Fig. 4A), the onset of the instability produces azimuthal phase trapping characteristic of ECMI (Fig. 4B) and efficiently amplifies the harmonics (Fig. 4F). At the point of classical saturation, the ring continues to contract and is sustained, as evidenced by the smaller radius (Fig. 4C), and the frequency of each harmonic undergoes a slight upshift (evident in Figs. 3A and 4G).

The phase trapping caused by the amplification process radially widens the ring, but this is counterbalanced by the bunching effect of radiative cooling, leading to a broader yet still well-defined ring (Fig. 4D). Consequently, the electromagnetic spectrum broadens at late times (Figs. 3A and 4H), indicating that the nonlinear stage does not entirely inhibit population inversion. The ring structure in momentum space remains intact, and the ECMI continues to be active well beyond the timescales presented in this work (Fig. 3D).

Eventually, the ring will cool down to $T∼m_{e}c^{2}/\sqrt{3}$, the instability overcomes the bunching process [as the synchrotron losses which sustain the ring become less important (*28*)], and the ring is diffused, stopping the ECMI and emission process. This occurs when the ring cools down to a ring radius $p_{R}∼m_{e}c/\sqrt{3}$, which determines for how long the ring structure and emission can be sustained. In the plasma proper frame that is comparable to $t_{\text{em}}≃2\omega_{\text{ce}}^{-1}/\sqrt{3}\alpha B_{0}$, $t_{\text{em}}\left(400\;\mu s\right)≃{\left[B\left(\text{MG}\right)\right]}^{-2}$, which is independent of the plasma parameters, as it is a timescale determined solely by the cooling process when $t_{\text{em}}>t_{o}$.

Therefore, after the onset of the maser at $t_{o}$ (Eq. 2), the ring and the ECMI will be sustained until $t_{\text{em}}$, producing a long pulse of radiation. This finding addresses a major criticism of the ECMI as a source of “long-lived” coherent radiation (*41*). In relativistic plasmas, the onset of the maser instability does not disrupt the population inversion, allowing continued emission. Radiative effects sustain the population inversion, enabling the maser to operate over prolonged periods. Depending on the plasma parameters, the ring can form and cool below $p_{R}∼m_{e}c/\sqrt{3}$ before the onset of the ECMI, and, in that case, $t_{o}>t_{\text{em}}$. In this scenario, the onset of the ECMI can still happen, and the ring will begin to diffuse right after saturation. Radiative losses will not be able to reform or sustain the ring, resulting in a single pulse of electromagnetic waves that escapes the plasma, resembling the classical ECMI emission.

## DISCUSSION

The necessary plasma parameters for relativistic plasmas to emit coherent radiation via ECMI can be determined by guaranteeing that a hierarchy of timescales is fulfilled. First, the onset of the instability $t_{o}$ (Eq. 2) must be shorter than any diffusive process, e.g., Coulomb collisions, Compton collisions, and pair annihilation (see the Supplementary Materials). In addition, the onset time must be shorter than $t_{\text{landau}}≃\frac{2}{3\alpha }B_{0}^{-2}\omega_{\text{ce}}^{-1}$, i.e., the time it takes the ring to cool to the the lowest quantum Landau level when the plasma becomes degenerate.

**Fig. 5.**
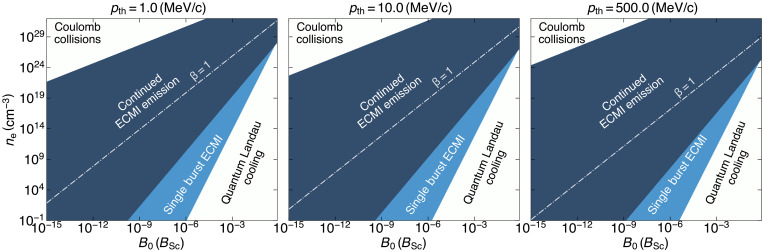
The comparison of relevant timescales for the onset of the synchrotron ECMI demonstrates that maser emission occurs for a wide range of plasma and magnetic field parameters. As presented across three panels, each corresponding to different initial thermal energies and defined by plasma density, magnetic field strength, and initial thermal energy. Regions in dark blue indicate where the maser instability triggers and sustains continued emission, occurring before competing processes such as collisional relaxation or cooling to quantum Landau levels can interfere. The top left white region highlights conditions where collisional relaxation dominates, diffusing the ring structure and preventing efficient maser emission, while the right-hand white region shows where cooling to quantum Landau levels occurs before the maser instability can develop, rendering the plasma degenerate and preventing maser onset. A light blue region represents conditions where the maser instability triggers with a single burst produced, as the onset occurs after the ring has cooled below the threshold thermal momentum needed for sustained bunching. A white dashed line marks the boundary where the plasma beta parameter β, i.e., the ratio of plasma pressure to magnetic field pressure, is β=1. Above this line, other instabilities, such as the synchrotron firehose instability, can also trigger.

There is a wide range of parameters for which the synchrotron-induced ECMI operates efficiently before (Coulomb or Compton) collisional relaxation and cooling to the quantum levels can take place (see Fig. 5). The parameter space increases as the plasma becomes more energetic. The ECMI will spontaneously trigger in a wide range of plasma parameters of relevance to astrophysical emissions and observations (*33*). Other instabilities can also trigger because of synchrotron losses; the firehose instability can operate when β>1, where β is the ratio of the plasma pressure to the magnetic field pressure (*27*). In that regime, the firehose instability will modify the momentum distribution, but as the plasma cools down, the plasma can transition to a β<1 regime enabling a modified version of the “ring” ECMI to operate. Preliminary PIC simulations have confirmed this picture and will be explored elsewhere.

The transmission efficiency at a sharp plasma boundary, $\sqrt{\varepsilon \left(\omega \right)}$, is determined by the plasma permittivity ε(ω) (*39*). Because the fastest-growing mode, the linearly polarized X-mode, has a wave frequency on the order of $\omega_{\text{ce}}$ in a tenuous, highly magnetized plasma ($\omega_{\text{pe}}\ll \omega_{\text{ce}}$), we approximate $\varepsilon \left(\omega \right)≃1-\omega_{\text{pe}}^{2}/\omega^{2}$ (*42*). For the parameters studied, where $\omega_{\text{pe}}∼0.002\;\omega_{\text{ce}}$ and $\omega ∼\omega_{\text{ce}}$, the resulting transmission coefficient is $\sqrt{\varepsilon \left(\omega \right)}∼1$. While specific plasma profiles, such as density gradients or inhomogeneities, could lower the conversion efficiency, this estimate demonstrates that the effect of impedance mismatch is minimal. Thus, we conjecture that the conversion efficiency of X-modes into emitted light waves as they escape the plasma volume is remarkably high. This emission spans a wide frequency range, depending on the magnetic field parameters, and includes radio frequencies. In the proper frame of the plasma, the emitted frequencies occur at harmonics of $n\omega_{\text{ce}}/\gamma_{r}$, where $\gamma_{r}={\left(1+p_{r}^{2}/m_{e}^{2}c^{2}\right)}^{1/2}$ is the Lorentz factor associated with the ring radius at the onset of the maser instability. Radio emission occurs in the proper frame at $\omega \;\left(17\;\text{THz}\right)≃B\;\left(\text{MG}\right)/\gamma_{r}\;\left(1\right)$. In addition, the emission maintains a constant ratio of Δω/ω, where Δω is the frequency separation between different harmonics. This constant ratio is observed in sources such as the Crab pulsar (*43*).

Using the largest PIC simulations to date for tenuous, synchrotron-cooled plasmas, we demonstrated that these plasmas can spontaneously produce coherent radiation when self-consistent electrodynamical radiative effects are considered. This radiation is driven by the onset of the ring-driven ECMI. Our results reveal that this emission process can persist for substantially longer periods than previously thought because of the interplay between the instability and synchrotron losses. This finding challenges the classical understanding of ECMI, which has traditionally been seen as resulting in only short bursts of radiation due to rapid saturation, and demonstrates the relevance of ECMI in synchrotron-cooled relativistic plasmas.

Our findings suggest that the synchrotron-driven ECMI is relevant beyond the specific plasma conditions explored in our simulations, having also a broader applicability across various plasma and magnetic field parameters. The timescales and properties of this fundamental plasma process were estimated using an idealized electromagnetic field configuration, i.e., a constant magnetic field in the proper frame of an arbitrary beam. Previous work has explored the limiting conditions for ring formation in curved and inhomogeneous magnetic fields (*28*) and found them to remain compatible with astrophysical conditions, including the curved or inhomogeneous fields expected near astrophysical objects, as well as modest thermal spreads. Future work will incorporate these nonideal electromagnetic field configurations into self-consistent simulations, which may modify emission properties and, in extreme cases, partially or fully suppress emission. Nonetheless, preliminary estimates suggest that ring formation remains robust under these conditions (*28*).

This implies that this mechanism can operate in a wide range of astrophysical environments. Notably, this mechanism appears to align with several key features observed in pulsar and magnetar emissions (*44*), offering a potential explanation for certain characteristics of fast radio bursts (FRBs) and pulsar emission (*45*, *46*). The observed connection between FRBs and magnetars, particularly the detection of FRBs coinciding with magnetar glitches, suggests a model where pair plasmas in a low-twist magnetar magnetosphere generate these bursts (*2*, *47*, *48*). Our findings demonstrate that synchrotron losses combined with the ECMI can sustain coherent emission over longer durations, which may explain the observed coherence, radio emission range, linear polarization, repetition, and similarities across diverse astronomical objects. This mechanism is a result of the unique qualitative properties of extreme plasmas. The resulting synchrotron-induced ECMI mechanism has implications that extend beyond the specific case of FRBs to a broader spectrum of astrophysical phenomena and future astro-laboratory experiments.

## MATERIALS AND METHODS

### PIC simulations

We study radiatively cooled rings and the subsequent ECMI via PIC simulations with OSIRIS (*49*), including classical (*50*) and QED (*51*) radiation reaction. The PIC method is widely used in plasma physics to model the behavior of plasmas by solving the equations of motion for charged particles and the self-consistent evolution of electromagnetic fields. The plasma is represented by a large number of particles, which move according to the Lorentz force [with Landau-Lifshiftz force to account for semiclassical radiative losses (*35*, *50*) and QED Monte Carlo module to account for QED processes (*51*)] in response to the electromagnetic fields. These fields, in turn, are computed on a grid using Maxwell’s equations, with the particle motions and fields updated iteratively.

PIC simulations are well suited for studying kinetic instabilities in plasmas, such as the ECMI, because they capture the full range of particle interactions and nonlinear effects. The massively parallel nature of these simulations allows for the handling of large-scale problems, making it possible to explore complex phenomena in tenuous, synchrotron-cooled plasmas with high fidelity and at unprecedented scales.

For the simulations presented in this work, we considered the setup described analytically. The ECMI is a kinetic instability for which the relevant dynamics occur in momentum space, and the resulting excited wave modes propagate either parallel or perpendicular to the magnetic field. Therefore, our simulations use a two-dimensional (2D) configuration space and full 3D momentum space. This guarantees that all the relevant physics of the ECMI and cooling dynamics are captured in our setup. There is a magnetic field aligned along the $x_{1}$ direction of strength B=100 GG, i.e., $B_{0}≃0.002$ normalized to the Schwinger field ($B_{\text{Sc}}=4.4\times 10^{9}\;T$). The magnetic field has a cyclotron frequency $\omega_{\text{ce}}=|e|B/m_{e}=1.75\times 10^{18}\;\text{Hz}$. All relevant timescales and lengths are normalized to the cyclotron period $\omega_{\text{ce}}^{-1}$ and $c/\omega_{\text{ce}}$. The simulations use a small time step such that the cyclotron period is accurately resolved $\Delta t=0.014\;\omega_{\text{ce}}^{-1}$ and a spatial resolution of $\Delta x=0.02\;c/\omega_{\text{ce}}$ (in both directions), which fulfils the 2D Courant condition $\Delta x>2^{-1/2}c\Delta t$. This resolution resolves the gyromotion of all electrons with at least ~70 temporal steps, with higher energy particles being resolved with even more spatial and temporal points. The simulation window has a size of $L_{1}=200\;c/\omega_{\text{ce}}$ and $L_{2}=1000\;c/\omega_{\text{ce}}$, this yields a simulation grid of size 10,000×50,000, and we use 16 particles per cell, i.e., a total of 16 billion computational particles.

The dimensions of the simulation box were carefully chosen, the $x_{2}$ direction uses a larger domain to capture the theoretically predicted modes that propagate perpendicular and almost perpendicular to the magnetic field (a 1D3V PIC simulation would solely capture the the wave dynamics propagating perfectly parallel to the simulation domain but not those at small angles).

The simulations were run for $40,000\;\omega_{\text{ce}}^{-1}$, and this is $∼2.8\times 10^{6}$ time iterations. Because of the size of each simulation, the high-resolution runs were performed in LUMI-C (Finland) and had an average cost of 1 million central processing unit hours. Smaller simulations were performed in Deucalion (Portugal).

The plasma was initialized with a plasma frequency ratio of $\omega_{\text{pe}}/\omega_{\text{ce}}≃0.00223$ for each species. This value was carefully chosen to create a low-density electron-positron pair plasma within the simulation domain, using periodic boundary conditions. The simulation encompasses several skin depths and Debye lengths throughout the cooling process and the development of the instability, effectively modeling a tenuous pair plasma in a strong magnetic field where $\omega_{\text{pe}}/\omega_{\text{ce}}\ll 1$. The pair plasma is initialized from a Maxwellian momentum distribution $f\left(p_{⊥},p_{∥}\right)\propto e^{-\left(p_{⊥}^{2}+p_{∥}^{2}\right)/\left(2p_{\text{th}}^{2}\right)}$, with $p_{\text{th}}=1000\;m_{e}c$. Simulations with a Maxwell-Jüttner distribution were also performed and, as expected, provided the same numerical results.

The macroparticles use cubic interpolation. Different current smoothing filters were tested, and we found that first-order binomial smoothing was sufficient to reduce the computational collisionality for the large number of time steps in the simulations. For the simulations shown in this work, OSIRIS used the Landau-Lifshitz model for classical radiation reaction as described in (*50*). Moreover, QED simulations, which use a Monte Carlo method to model quantum synchrotron emission (*51*), were also used, and QED simulations agree with simulations using the Landau-Lifshitz pusher with the inclusion of stochastic diffusion, as expected as $\chi ={\mathrm{pB}}_{0}/\left(m_{e}c\right)$ decreases rapidly during the cooling process. These effects will be explored elsewhere at higher energies, where diffusive effects are expected to be dominant.

Convergence studies were performed to determine the computational parameters and to ensure energy conservation accounting for synchrotron losses. All results, both numerical and analytical, are presented in the proper frame of the plasma or beam. This means that the results can be directly applied to beam-plasma systems in other reference frames through the appropriate Lorentz transformation.

### Degree of polarization of the maser radiation

The Stokes parameters and degree of polarization can be obtained from the PIC simulations presented in this work. For an observer with a line of sight along ${\hat{x}}_{2}$, perpendicular to the magnetic field along ${\hat{x}}_{1}$, the Stokes parameters in the Jones basis are given by (*52*)

$$I=\langle E_{1}^{2}\rangle +\langle E_{3}^{2}\rangle$$ (3)

$$Q=\langle E_{1}^{2}\rangle -\langle E_{3}^{2}\rangle$$ (4)

$$U=\langle E_{a}^{2}\rangle -\langle E_{b}^{2}\rangle$$ (5)

$$V=\langle E_{r}^{2}\rangle -\langle E_{l}^{2}\rangle$$ (6)

where the subscripts *a* and *b* represent the electric field components projected onto the cartesian basis rotated 45°. The subscripts *l* and *r* are the projection in the circular basis such that $\hat{l}=\left({\hat{x}}_{1}+i{\hat{x}}_{3}\right)/\sqrt{2}$ and $\hat{r}=\left({\hat{x}}_{1}-i{\hat{x}}_{3}\right)/\sqrt{2}$. The $<E_{i}^{2}>$ represents the averaged quantity such that $\langle E_{i}^{2}\rangle =\frac{1}{T}\int_{0}^{T}E_{i}^{2}\left(t\right)\mathrm{dt}$, where $E_{i}^{2}\left(t\right)$ is the square of the electric field at a given time at the observer’s position. From the simulation results, we can construct the different quantities in their respective Jones basis and average them spatially to synthetically obtain the Stokes parameters an observer would measure.

The degree of linear polarization is then defined as (*52*)

$$\Pi =\sqrt{Q^{2}+U^{2}}/I$$ (7)

The result from this synthetic diagnostic for the percentile degree of polarization, in the PIC simulations, is shown in Fig. 3B. Initially, the electromagnetic radiation is mostly unpolarized; later, once the onset of the ECMI ($t∼6000\;\omega_{\text{ce}}^{-1}$), the radiation becomes highly linearly polarized reaching a maximum Π=99.8%.

## Supplementary Materials

### This PDF file includes:

Supplementary Text Fig. S1 References
