# Supplementary material for: Radiative cooling induced coherent maser emission in relativistic plasmas
Source: Sci Adv. 2025 Apr 11;11(15):eadt8912. doi: 10.1126/sciadv.adt8912 (PMC11988401; doi:10.1126/sciadv.adt8912)
Supplement: Supplementary file 1 — Supplementary Text Fig. S1 References [file sciadv.adt8912_sm.pdf]

Science Advances
AAAS

Supplementary Materials for

Radiative cooling induced coherent maser emission in relativistic plasmas

Pablo J. Bilbao *et al.*

Corresponding author: Pablo J. Bilbao, pablojbilbao@tecnico.ulisboa.pt; Luis O. Silva, luis.silva@tecnico.ulisboa.pt

Sci. Adv. **11**, eadt8912 (2025)
DOI: 10.1126/sciadv.adt8912

This PDF file includes:

Supplementary Text
Fig. S1
References

Parameter scan of the onset time of the ECMI

In order to demonstrate the validity of the scaling of the onset time (*i.e.* Eq. (2)) a set of PIC simulations were performed while varying the key parameters. The results of this simulation campaign (displayed in Fig. S1) confirm our understanding of the tiemscales and onset of the instability.

The simulations performed are 1D3V, with the magnetic field being aligned perpendicular to the x_1-direction. This allows the propagation of X-mode waves with k perpendicular to B. The parameter scan confirms that the onset time in cyclotron periods scales as $t_o \propto p_{th}^{-1/2}\omega_{pe}^{-1}$. Moreover, an extra paramer scan was performed varying the magnetic field while keeping ω_{ce}/ω_{pe} constant. In doing so, as B_0 increases so does ω_{pe}. As $t_o \propto B_0^{1/2}/\omega_{pe}$, the resulting dependence is that $t_o \propto B_0^{-1/2}$ From the energy stored in the electromagnetic component E_3, *i.e.*, the electric field associated with the X-mode. We can determine the time onset of the instability when the change in the slope of the energy over time is significant when compared with the maximum energy achieved, the fitting code checks for the condition $\Delta E^2/\Delta t > 0.001E_{\max}^2$.

Timescales for collisional relaxation

We consider three relevant collisional processes that can diffuse the ring distribution before the onset of the electron cyclotron maser instability (ECMI). These processes are: (i) Coulomb collisions, (ii) pair annihilation, and (iii) Compton scattering from synchrotron self-emission. The relaxation timescale for each process is defined as the inverse of its corresponding collision frequency.

i) The relaxation timescale due to Coulomb collisions is given by $t_{ee} = \frac{12\pi^{3/2}}{\sqrt{2}}\frac{\epsilon_0^2 m_e^2 c^3}{e^4}\frac{1}{n\ln\Lambda}$ (*28, 54, 55*), where n is the plasma density, $\ln\Lambda$ is the Coulomb logarithm, and, as we are dealing with relativistic plasmas, we have approximated $v_e \sim c$. Alternatively, $t_{ee} \simeq 5/(2\sigma_T cn)$ and $t_{ee}\,[\mathrm{s}] \simeq 1.25\times 10^{14}/(n\,[\mathrm{cm}^{-3}])$, where σ_T is the Thomson cross-section.

ii) For pair annihilation, the relaxation timescale is estimated using the cross-section for electron-positron collisions, which can be approximated by the Thomson cross-section σ_T (*56, 57*). In this case, the plasma is not simply diffused but rather "evaporates" as electron-positron pairs annihilate and are converted into high-energy photons. This process removes particles from the plasma, leading to its gradual depletion. The timescale for this "evaporation" process is $t_{eva} = 1/(2\sigma_T cn)$.

We note that this timescale is comparable to t_{ee}.

iii) The relaxation timescale due to Compton scattering, induced by synchrotron self-emission, can also be estimated. For the purpose of this discussion, an overestimate of the collisional effects suffices. The collisional frequency is defined as $\nu_{e\gamma} = 2c\sigma_T n_\gamma$, where n_γ is the photon density. The photon density can be estimated as the energy budget divided by the average energy per photon, *i.e.* $n_\gamma = \Delta E/\hbar\langle\omega\rangle$, where ΔE is the change in energy of the electron population as it cools, $\langle\omega\rangle$ is the average photon angular frequency, and $\hbar$ is the reduced Planck constant. The change in energy in the electron population is $\Delta E = p_{th}^2 n_e \tau/(1 + p_{th}\tau)$, obtained from the equations of motion (*26, 28*), where $\tau = \frac{2}{3}\alpha B_o t \omega_{ce}^{-1}$ with t being the time elapsed since the beginning of the cooling, and p_{th} is the initial thermal spread. We assume the average frequency $\langle\omega\rangle$ to be the critical frequency for a given ring radius at time t_o, which may underestimate the actual average frequency since electrons have higher energies earlier in the process. This overestimates the photon density at time t. This can be used to estimate the photon density and subsequently the relaxation time due to synchrotron self-emission at the onset time t_o. $t_{e\gamma} = 1/(2\sigma_T p_{\mathrm{th}}^{1/3} B_0 n_e^{1/3} n_o^{2/3})$, where $n_o = 596488\,\mathrm{cm}^{-3}$, and $t_{e\gamma}\,[3.5\times10^8\,\mathrm{s}] = B_0^{-1}(n_e\,[\mathrm{cm}^{-3}]\; p_{th}\,[m_e c])^{-1/3}$. This estimate demonstrates that before the onset of the ECMI, the the timescale of relaxation due to compton self-synchrotron scattering is much larger than Coulomb collisions, unless the plasma is highly magnetised or the thermal energy of the distribution is much higher than the parameter space we are interested in.

In this estimate, we have neglected the non-linear Breit-Wheeler mechanism, where photons convert into pairs. This reduces the photon density, which would lengthen the relaxation timescale. Moreover, while the plasma is large, high-energy photons can have mean free paths longer than the plasma's spatial scale, allowing some photons to escape without contributing to plasma relaxation. For further details on the balance between self-absorption and emission, refer to Ochs (2024) (*29*).

By comparing these timescales against the onset time, we obtain the parameter space in which the maser can operate, as shown in Fig. 5 of the main text.

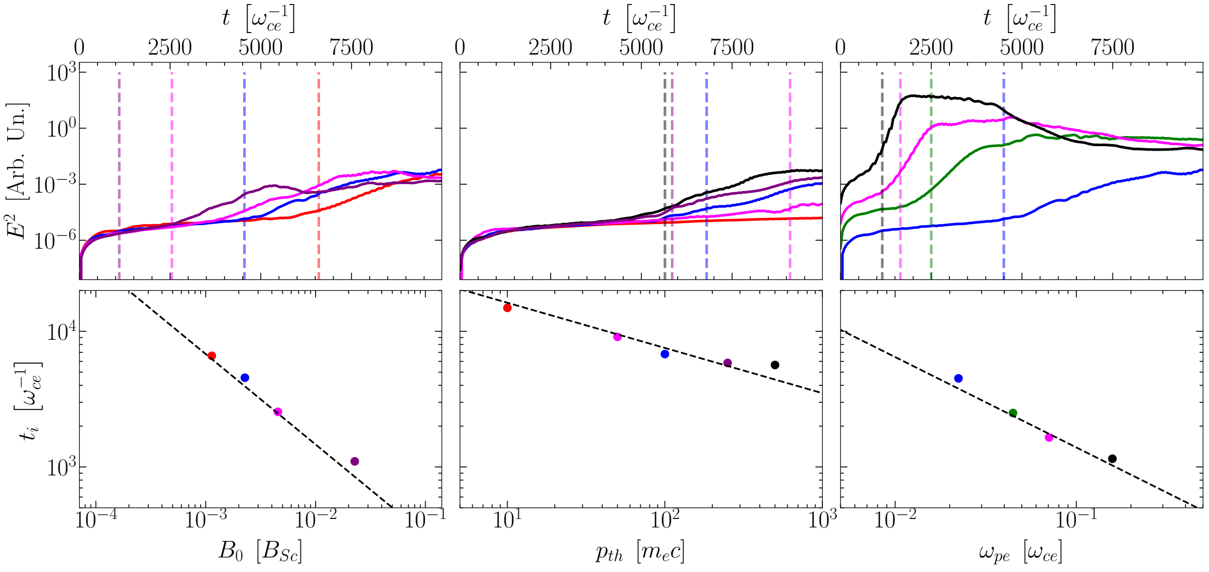

Figure S1:

Caption Supplementary Figure 1: Particle-in-cell simulation results demonstrate the correlation between the onset of electron cyclotron maser instability and the relevant parameters. The top row depicts the field energy for different simulations, each varying a single parameter per column. In the bottom row, the onset time for each simulation is presented in a log-log scale, showcasing its dependence on the varying parameter. The first column shows the results from varying the magnetic field $B_0 = B/B_{Sc}$. The second column shows the results from a varying initial thermal spread p_{th}. The last column shows the results from varying the plasma frequency ω_{pe}. The simulation outcomes are compared against the expected dependencies from Eq. (8) in the main text. Dashed lines represent the expected dependencies on B_0, p_{th}, and ω_{pe}, which are $t_o \propto B_0^{-1/2}$ (for a fixed (ω_{pe}/ω_{ce} ratio)), and$t_o \propto p_{th}^{-1/2}\,\omega_{pe}^{-1}$, respectively.

REFERENCES AND NOTES

1. P. Goldreich, W. H. Julian, Pulsar electrodynamics. *Astrophys. J.* **157**, 869 (1969).

2. A. N. Timokhin, A. K. Harding, On the polar cap cascade pair multiplicity of young pulsars. *Astrophys. J.* **810**, 144 (2015).

3. A. A. Philippov, A. Spitkovsky, B. Cerutti, Ab initio pulsar magnetosphere: Three-dimensional particle-in-cell simulations of oblique pulsars. *Astrophys. J. Lett.* **801**, L19 (2015).

4. A. Levinson, B. Cerutti, Particle-in-cell simulations of pair discharges in a starved magnetosphere of a Kerr black hole. *Astronom. Astrophys.* **616**, A184 (2018).

5. F. Cruz, T. Grismayer, A. Y. Chen, A. Spitkovsky, L. O. Silva, Coherent emission from QED cascades in pulsar polar caps. *Astrophys. J. Lett.* **919**, L4 (2021).

6. G. Sarri, K. Poder, J. M. Cole, W. Schumaker, A. Di Piazza, B. Reville, T. Dzelzainis, D. Doria, L. A. Gizzi, G. Grittani, S. Kar, C. H. Keitel, K. Krushelnick, S. Kuschel, S. P. D. Mangles, Z. Najmudin, N. Shukla, L. O. Silva, D. Symes, A. G. R. Thomas, M. Vargas, J. Vieira, M. Zepf, Generation of neutral and high-density electron–positron pair plasmas in the laboratory. *Nat. Commun.* **6**, 6747 (2015).

7. T. Grismayer, M. Vranic, J. L. Martins, R. Fonseca, L. Silva, Laser absorption via quantum electrodynamics cascades in counter propagating laser pulses. *Phys. Plasmas* **23**, 056706 (2016).

8. C. Zhang, C.-K. Huang, K. A. Marsh, C. E. Clayton, W. B. Mori, C. Joshi, Ultrafast optical field–ionized gases—A laboratory platform for studying kinetic plasma instabilities. *Sci. Adv.* **5**, eaax4545 (2019).

9. H. Chen, F. Fiuza, Perspectives on relativistic electron–positron pair plasma experiments of astrophysical relevance using high-power lasers. *Phys. Plasmas* **30**, 020601 (2023).

10. C. D. Arrowsmith, P. Simon, P. J. Bilbao, A. F. A. Bott, S. Burger, H. Chen, F. D. Cruz, T. Davenne, I. Efthymiopoulos, D. H. Froula, A. Goillot, J. T. Gudmundsson, D. Haberberger, J. W. D. Halliday, T. Hodge, B. T. Huffman, S. Iaquinta, F. Miniati, B. Reville, S. Sarkar, A. A.

Schekochihin, L. O. Silva, R. Simpson, V. Stergiou, R. M. G. M. Trines, T. Vieu, N. Charitonidis, R. Bingham, G. Gregori, Laboratory realization of relativistic pair-plasma beams. *Nat. Commun.* **15**, 5029 (2024).

11. K. Qu, A. Griffith, N. J. Fisch, Pair filamentation and laser scattering in beam-driven QED cascades. *Phys. Rev. E* **109**, 035208 (2024).

12. E. E. Los, E. Gerstmayr, C. Arran, M. J. V. Streeter, C. Colgan, C. C. Cobo, B. Kettle, T. G. Blackburn, N. Bourgeois, L. Calvin, J. Carderelli, N. Cavanagh, S. J. D. Dann, A. DiPiazza, R. Fitzgarrald, A. Ilderton, C. H. Keitel, M. Marklund, P. McKenna, C. D. Murphy, Z. Najmudin, P. Parsons, P. P. Rajeev, D. R. Symes, M. Tamburini, A. G. R. Thomas, J. C. Wood, M. Zepf, G. Sarri, C. P. Ridgers, S. P. D. Mangles, Observation of quantum effects on radiation reaction in strong fields. arXiv:2407.12071 [hep-ph] (2024).

13. A. Di Piazza, K. Hatsagortsyan, C. Keitel, Strong signatures of radiation reaction below the radiation-dominated regime. *Phys. Rev. Lett.* **102**, 254802 (2009).

14. A. Thomas, C. Ridgers, S. Bulanov, B. Griffin, S. Mangles, Strong radiation-damping effects in a gamma-ray source generated by the interaction of a high-intensity laser with a wakefield-accelerated electron beam. *Phys. Rev. X* **2**, 041004 (2012).

15. M. Vranic, J. L. Martins, J. Vieira, R. A. Fonseca, L. O. Silva, All-optical radiation reaction at 10^{21} W/cm^2. *Phys. Rev. Lett.* **113**, 134801 (2014).

16. V. M. Kaspi, A. M. Beloborodov, Magnetars. *Annu. Rev. Astron. Astrophys.* **55**, 261–301 (2017).

17. B. Cerutti, A. M. Beloborodov, Electrodynamics of pulsar magnetospheres. *Space Sci. Rev.* **207**, 111–136 (2017).

18. V. Zhdankin, D. A. Uzdensky, G. R. Werner, M. C. Begelman, Kinetic turbulence in shining pair plasma: Intermittent beaming and thermalization by radiative cooling. *Mon. Not. R. Astron. Soc.* **493**, 603–626 (2020).

19. L. Comisso, L. Sironi, Pitch-angle anisotropy controls particle acceleration and cooling in radiative relativistic plasma turbulence. *Phys. Rev. Lett.* **127**, 255102 (2021).

20. M. Zhou, V. Zhdankin, M. W. Kunz, N. F. Loureiro, D. A. Uzdensky, Magnetogenesis in a collisionless plasma: From Weibel instability to turbulent dynamo. *Astrophys. J.* **960**, 12 (2024).

21. I. Plotnikov, L. Sironi, The synchrotron maser emission from relativistic shocks in fast radio bursts: 1D PIC simulations of cold pair plasmas. *Mon. Not. R. Astron. Soc.* **485**, 3816–3833 (2019).

22. A. Vanthieghem, J. F. Mahlmann, A. Levinson, A. Philippov, E. Nakar, F. Fiuza, The role of plasma instabilities in relativistic radiation-mediated shocks: Stability analysis and particle-in-cell simulations. *Mon. Not. R. Astron. Soc.* **511**, 3034–3045 (2022).

23. S. V. Bulanov, G. M. Grittani, R. Shaisultanov, T. Z. Esirkepov, C. P. Ridgers, S. S. Bulanov, B. K. Russell, A. G. R. Thomas, On the energy spectrum evolution of electrons undergoing radiation cooling. *Fundam. Plasma Phys.* **9**, 100036 (2024).

24. K. Qu, S. Meuren, N. J. Fisch, Signature of collective plasma effects in beam-driven QED cascades. *Phys. Rev. Lett.* **127**, 095001 (2021).

25. D. Uzdensky, M. Begelman, A. Beloborodov, R. Blandford, S. Boldyrev, B. Cerutti, F. Fiuza, D. Giannios, T. Grismayer, M. Kunz, N. Loureiro, M. Lyutikov, M. Medvedev, M. Petropoulou, A. Philippov, E. Quataert, A. Schekochihin, K. Schoeffler, L. Silva, L. Sironi, A. Spitkovsky, G. Werner, V. Zhdankin, J. Zrake, E. Zweibel, Extreme plasma astrophysics. arXiv:1903.05328 [astro-ph.HE] (2019).

26. P. J. Bilbao, L. O. Silva, Radiation reaction cooling as a source of anisotropic momentum distributions with inverted populations. *Phys. Rev. Lett.* **130**, 165101 (2023).

27. V. Zhdankin, M. W. Kunz, D. A. Uzdensky, Synchrotron firehose instability. *Astrophys. J.* **944**, 24 (2023).

28. P. J. Bilbao, R. J. Ewart, F. Assunçao, T. Silva, L. O. Silva, Ring momentum distributions as a general feature of Vlasov dynamics in the synchrotron dominated regime. *Phys. Plasmas* **31**, 052112 (2024).

29. I. E. Ochs, Synchrotron-driven instabilities in relativistic plasmas of arbitrary opacity. arXiv:2407.13106 [physics.plasm-ph] (2024).

30. P. Sprangle, A. Drobot, The linear and self-consistent nonlinear theory of the electron cyclotron maser instability. *IEEE Trans. Microw. Theory Tech.* **25**, 528–544 (1977).

31. K.-R. Chen, J. M. Dawson, A. T. Lin, T. Katsouleas, Unified theory and comparative study of cyclotron masers, ion-channel lasers, and free electron lasers. *Phys. Fluids B* **3**, 1270–1278 (1991).

32. R. Bingham, R. Cairns, Generation of auroral kilometric radiation by electron horseshoe distributions. *Phys. Plasmas* **7**, 3089–3092 (2000).

33. D. Melrose, Coherent emission mechanisms in astrophysical plasmas. *Rev. Mod. Plasma Phys.* **1**, 1–81 (2017).

34. R. A. Treumann, The electron–cyclotron maser for astrophysical application. *Astron. Astrophys. Rev.* **13**, 229–315 (2006).

35. L. D. Landau, E. M. Lifshitz, *The Classical Theory of Fields*, (Pergamon Press, 1975), vol. 2.

36. L. Kuz'menkov, The Bogolyubov hierarchy of equations for relativistic systems. Radiation damping of waves in a plasma. *Sov. Phys. Dokl.* **23**, 469–471 (1978).

37. R. J. Ewart, A. Brown, T. Adkins, A. A. Schekochihin, Collisionless relaxation of a Lynden-Bell plasma. *J. Plasma Phys.* **88**, 925880501 (2022).

38. M. L. Nastac, R. J. Ewart, W. Sengupta, A. A. Schekochihin, M. Barnes, W. D. Dorland, Phase-space entropy cascade and irreversibility of stochastic heating in nearly collisionless plasma turbulence. *Phys. Rev. E* **109**, 065210 (2024).

39. A. F. Alexandrov, L. S. Bogdankevich, A. A. Rukhadze, *Principles of Plasma Electrodynamics* (Springer, 1984), vol. 9.

40. R. Winglee, Fundamental and harmonic electron cyclotron maser emission. *J. Geophys. Res. Space Physics* **90**, 9663–9674 (1985).

41. W. Lu, P. Kumar, On the radiation mechanism of repeating fast radio bursts. *Mon. Not. R. Astron. Soc.* **477**, 2470–2493 (2018).

42. T. H. Stix, *Waves in Plasmas* (Springer Science & Business Media, 1992).

43. T. Hankins, G. Jones, J. Eilek, The Crab pulsar at centimeter wavelengths. I. Ensemble characteristics. *Astrophys. J.* **802**, 130 (2015).

44. A. Philippov, M. Kramer, Pulsar magnetospheres and their radiation. *Annu. Rev. Astron. Astrophys.* **60**, 495–558 (2022).

45. M. Bailes, The discovery and scientific potential of fast radio bursts. *Science* **378**, eabj3043 (2022).

46. W. Zhu, H. Xu, D. Zhou, L. Lin, B. Wang, P. Wang, C. Zhang, J. Niu, Y. Chen, C. Li, L. Meng, K. Lee, B. Zhang, Y. Feng, M. Ge, E. Göğüş, X. Guan, J. Han, J. Jiang, P. Jiang, C. Kouveliotou, D. Li, C. Miao, X. Miao, Y. Men, C. Niu, W. Wang, Z. Wang, J. Xu, R. Xu, M. Xue, Y. Yang, W. Yu, M. Yuan, Y. Yue, S. Zhang, Y. Zhang, A radio pulsar phase from SGR J1935+2154 provides clues to the magnetar FRB mechanism. *Sci. Adv.* **9**, eadf6198 (2023).

47. C. D. Bochenek, V. Ravi, K. V. Belov, G. Hallinan, J. Kocz, S. R. Kulkarni, D. L. McKenna, A fast radio burst associated with a Galactic magnetar. *Nature* **587**, 59–62 (2020).

48. CHIME/FRB Collaboration, Sub-second periodicity in a fast radio burst. *Nature* **607**, 256–259 (2022).

49. R. Fonseca, L. O. Silva, F. Tsung, V. Decyk, W. Lu, C. Ren, W. Mori, S. Deng, S. Lee, T. Katsouleas, J. Adam, OSIRIS: A three-dimensional, fully relativistic particle in cell code for

modeling plasma based accelerators, in *International Conference on Computational Science* (Springer, 2002), pp. 342–351.

50. M. Vranic, J. L. Martins, R. A. Fonseca, L. O. Silva, Classical radiation reaction in particle-in-cell simulations. *Comput. Phys. Commun.* **204**, 141–151 (2016).

51. M. Vranic, T. Grismayer, R. A. Fonseca, L. O. Silva, Quantum radiation reaction in head-on laser-electron beam interaction. *New J. Phys.* **18**, 073035 (2016).

52. G. B. Rybicki, A. P. Lightman, *Radiative Processes in Astrophysics* (John Wiley & Sons, 1991).

53. P. J. Bilbao, T. Silva, L. O. Silva, Simulation results: Radiative cooling induced coherent maser emission in relativistic plasmas (v1.0), Zenodo (2024); https://doi.org/10.5281/zenodo.13947842.

54. B. Trubnikov, Particle interactions in a fully ionized plasma. *Rev. Plasma Phys.* **1**, 105–140 (1965).

55. R. Goldston, P. Rutherford, *Introduction to plasma physics* (IOP Publishing, 1995).

56. A. Lightman, Relativistic thermal plasmas - Pair processes and equilibria. *Astrophys. J.* **253**, 842–858 (1982).

57. J. M. Jauch, F. Rohrlich, *The Theory of Photons and Electrons: The Relativistic Quantum Field Theory of Charged Particles with Spin One-Half* (Springer Science & Business Media, 2012).
